# Gully erosion is a serious obstacle in India’s land degradation neutrality mission

**DOI:** 10.1038/s41598-025-89613-w

**Published:** 2025-02-21

**Authors:** Anindya Majhi, Pritha Bhattacharjee, Angela Harris, Martin Evans, Emma Shuttleworth

**Affiliations:** 1 Department of Geography, School of Environment, Education and Development, The University of Manchester, Oxford Road, Manchester, M13 9PL UK; 2 Independent Geographer, Kalitala Park (South), Bansdroni, 700 070 Kolkata India; 3 Faculty of Social Sciences and Health, Durham University, Stockton Road, Durham, DH1 3LE UK

**Keywords:** India, Gully erosion, Gully dynamics, Badlands, Land management, Land degradation neutrality, Sustainability, Natural hazards

## Abstract

India is famous for her badlands. These vast, intensely degraded landscapes occur extensively across Central and Western India, wherein they have had several adverse effects on both environment and society. However, accurate information on their current spatial extents, as well as the spatial distribution and severity of gully erosion elsewhere in the country was hitherto lacking. Considering that India has planned to effectively halt land degradation by 2030 in line with the agenda of the United Nations, and as precise spatial data is indispensable in planning and implementing land management interventions, we have created an exhaustive spatial inventory of gully erosion features in India by recording their location, extents and current management status from high-resolution satellite imagery available on Google Earth Pro. Through this first of its kind mapping endeavour and attendant spatial analysis, we have recorded the presence of gully landforms in 19 of India’s 28 states and the National Capital Region of Delhi and have estimated the total gullied area in the country between 7,451 and 8,157 km^2^. According to our results, states occupying 38% of Indian territory (*viz.*, Rajasthan, Uttar Pradesh, Madhya Pradesh, Jharkhand, Gujarat and Chhattisgarh) are affected by 92% of the total gullied area of the country. We have noted a clear east-west divide in terms of the relative dominance of the mapped gully erosion features, with badlands being common in Western India and gully systems being the dominant gully feature in the east. A similar observation has interestingly also been made as regards gully management, with the major proportion of unmanaged gully erosion features occurring in Eastern India. Ultimately, we have identified 77 districts across India where urgent rehabilitative intervention is required, more than 70% of which are in Eastern and Southern India where unmanaged (active) gullies are common. That contemporary gully erosion in Eastern India poses a more serious land management challenge than that of the vast badlands of Central and Western India is a truly unexpected finding of our analysis. Our mapped data and analytical results shall be integral to efforts aiming to ameliorate the land degradation caused by gully erosion across India by supporting policymaking and planning at the governmental level and serving as useful guidance for land managers and practitioners on the ground.

**Supplementary Information:**

The online version contains supplementary material available at 10.1038/s41598-025-89613-w.

## Introduction

Gully erosion is a major driver of global land degradation^1^. Gullies occur where concentrated surface and/or subsurface runoff, possibly coupled with shallow mass movements, incises several tens of metres into the soil profile and carves out erosional channels^2,3^. Gully erosion is distinct from other types of soil erosion such as splash or interrill erosion by virtue of its deeply erosive nature and attendant higher specific soil losses^1^. Gully erosion is a particularly problematic type of land degradation, as its impact is not restricted to the land surface or the topsoil, its unpredictability, dynamic nature and the strong spatio-temporal variability^1,4,5^.

Gully erosion has several socio-environmental and geomorphological fallouts. It impairs in-situ soil quality^6,7^, depletes landscape carbon and heavy metal storage^8,9^, curtails crop yields and biomass^10,11^, spoils ex-situ water quality and reduces reservoir storage capacity^12,13^. The ability of gully erosion to enhance landscape connectivity^3,14^, alter catchment hydrological functioning^15 ^and to bring about drastic land use changes^16,17 ^is also significant. Gullies interact with other erosion processes and landslides^18,19 ^and drives long-term landscape evolution, by facilitating the creation of badlands^20,21 ^and other large erosional features^22,23^. Gullies are also becoming increasingly hazardous due to climate change effects^24 ^and directly threatening human lives, livelihoods and societies^25–27^.

Overall, gully erosion can potentially undermine efforts to realise nine of the 17 sustainable development goals (SDG), specifically SDGs 2 (Zero hunger), 6 (Clean water and sanitation), 13 (Climate action), 14 (Life below water) and 15 (Life on land). However, once a gully forms and is actively eroding, stabilising it through land management is often difficult to impossible^2,28^. Nevertheless, the cost of unmanaged gully erosion is often quite high, both to the local land users and downstream communities and environments^29,30^. The scientific underpinnings of gully remediation are well understood^29^, but accurate spatial information is critical in identifying regions in need of rehabilitative interventions and developing appropriate land management plans^31,32^.

Fortunately, contrary to other water erosion processes (*e.g*., piping or (inter)rill erosion), gullies are easily identifiable and mappable from remotely sensed imagery having an appropriately high (metre or submetre) spatial resolution^33–35^. Traditionally, such high horizontal resolutions have only been offered by aerial photographs and commercial satellite imagery, which have helped in spatial inventorying of gullies and understanding their dynamics, either through manual on-screen digitisation^36–40^, semi-automated image classification^41–45^ or repeat photogrammetry^46,47^. Considering that such very high resolution remotely sensed images are not usually openly accessible, the importance of Google Earth cannot be emphasised enough in this regard^48^. It is nowadays being used widely to develop gully (head) occurrence datasets that are fed into data-driven predictive mapping frameworks to obtain gully susceptibility assessments^49–52^. Although less frequently, Google Earth has also been used in systematic inventorying of gullies^53,54^, random mapping of gully occurrence^55–57^ and to quantify gully activity rates^58,59^.

India, an important country of the Global South (Population: 1.417 billion; Area: 3.287 million km^2^^2^), faces severe, and somewhat unique, gully erosion problems. Unlike any other country affected by gully erosion, badlands, which are extensive deeply-dissected landscapes formed due to prolonged and intense gully erosion, account for the majority share of gullied area in India^60,61^. The Indian badlands are closely associated with poor regional and socio-economic conditions^62,63^, particularly because they have caused agricultural productivity to dwindle, water scarcity and droughts to become common, entire villages to be abandoned and the landscape to become so fragmented and labyrinthine that it was an easy hideout for antisocial elements in the past^60^. The earliest gully mapping endeavours in India^64–66^ therefore solely focused on the delineation of badlands and identification of areas to be rehabilitated and reclaimed (based on gully depths and widths), as badlands management was a national policy priority^60^. Since the turn of the century, more studies have been conducted to identify the means to (semi-)automatically delineate the badlands from satellite images^67,68^, better understand their geomorphic and environmental characteristics from radar and/or optical (stereo) images^69–71^ and monitor their progressive reclamation^72,73^. The first pan-Indian gully erosion map (1:15 million), which was produced using (topographical) maps and information available in the literature^74^, is largely imprecise (due to the scale) for planning land management. It is a well-known fact that the badlands are especially widespread in central and western parts of the country^60^, but there exists no reliable information on their current spatial extents^31^. Furthermore, there is no accurate information on the spatial distribution and severity of gully erosion in India beyond the regions affected by the badlands^31^.

In 2019, India hosted the 14th session of the Conference of Parties to the United Nations Convention to Combat Desertification, and pledged to halt land degradation in the country as well as to rehabilitate 26 million hectares of degraded lands by 2030^75^^–^^77^, in line with the UN agenda of land degradation neutrality (LDN) by 2030 (SDG 15 target 3). As far as gully erosion is concerned, it is crucial to have a precise idea regarding its spatial distribution, areal extents and where it is (not) being rehabilitated, to inform prominent policymaking/planning discussions on gully management. Therefore, our overall aim is to create the first detailed spatial inventory of gully erosion in India through an unprecedented mapping of the location, extents and management conditions of gully erosion features using very high-resolution (≤1 m) satellite imagery. We subsequently use this data to evaluate the current status of gully management and estimate the total gullied land area across the two highest sub-national administrative levels, *i.e.*, states and districts. Using our results, we identify the provinces that are in need of rehabilitative intervention to tackle gully erosion and finally make a case for relevant policy (re)formulation to appropriately manage the gully erosion problem in India in the context of the national LDN drive.

## Methods

### Mapping the location of gully erosion features

We manually identified and mapped gully erosion features using the latest available (2020–2022) high-resolution (≤1 m) Quickbird, Worldview or Pleiades imagery on Google Earth Pro. A manual approach was preferred over automated image classification as high spatial resolution multispectral imagery spanning the territory of India are not freely available^60^, and automated mapping of gullies through spectral classification is known to be challenging due to significant variation in reflectance values from gullies, caused by differences in vegetation cover, soil organic matter and soil moisture^78,79^.

We employed the 15’ grid that is used for topographic mapping by the Survey of India^80^ as the foundation for creating the spatial inventory of gully erosion. This grid was used as it allowed us to consult the relevant topographic map and verify when gullies anywhere could not be identified with full confidence from the imagery (*i.e*., whether gullies or bedrock incisions). We subdivided each of the original 15’ grid cells into nine 5’ cells to facilitate the mapping on Google Earth Pro. To identify the mappable gully erosion features, each 5’ box was visually scanned from a viewing altitude of 1 km in a lawnmower pattern starting from the top left and navigating downwards, ensuring substantial scene overlap between adjacent scanning paths.

**Fig. 1:**
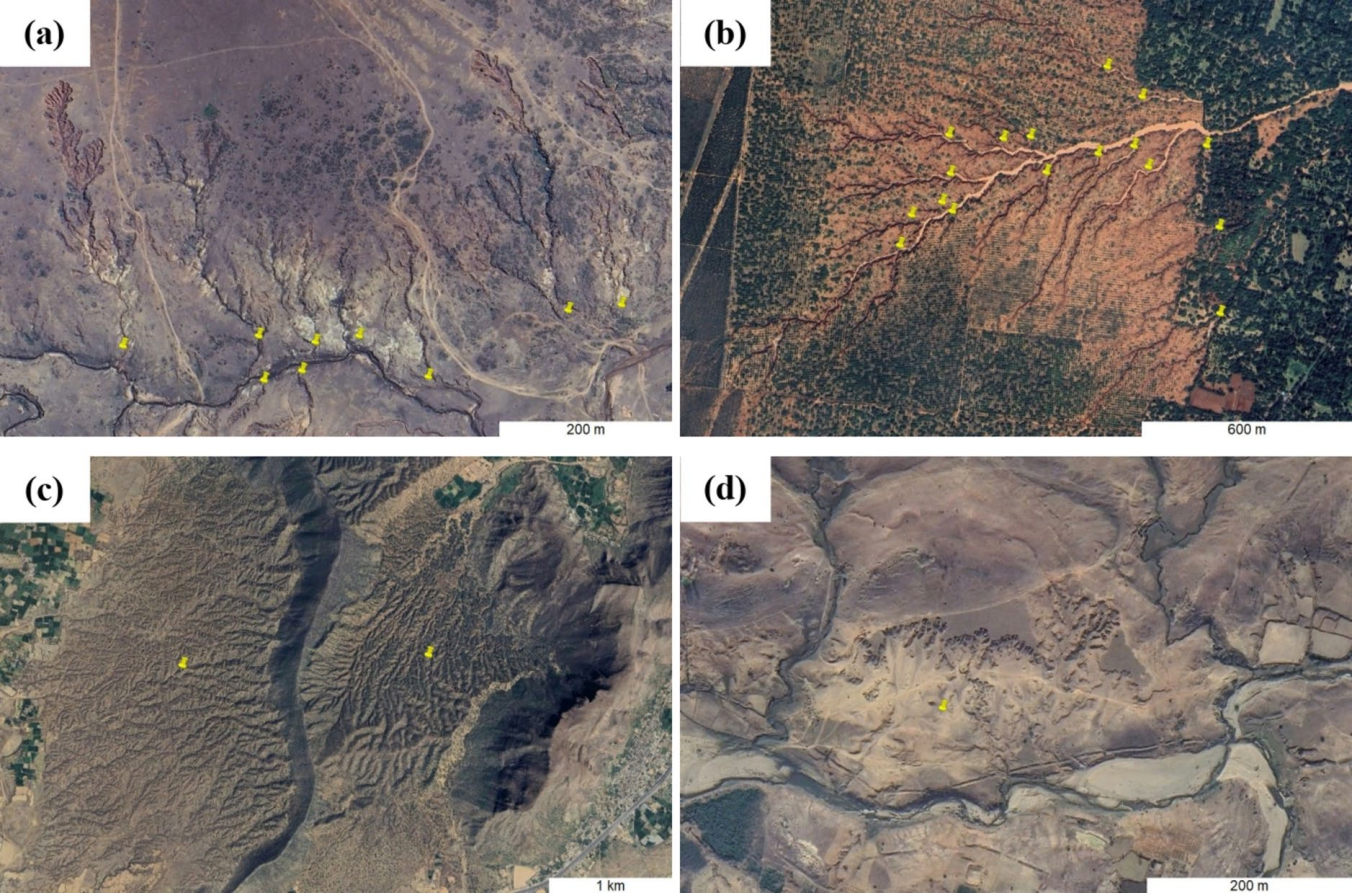
Mapping (a) hillslope gully systems terminating into a river, (b) headwater gully systems transitioning into an ephemeral channel, (c) badlands and (b) a denuded hillslope. *Note: Images exported from Google Earth Pro (2024 Airbus).*

In India, gully erosion manifests itself in three distinct geomorphic imprints: gully systems or gully networks (Fig. 1a and b), badlands (Fig. 1c) and denuded hillslopes (Fig. 1d). The location and type of each identified gully erosion feature was recorded using Google Earth Pro placemarks and associated description tags. For hillslope and bank gully systems draining into a river channel, the placemarks were dropped at the catchment outlet (Fig. 1a). For headwater gullies transitioning into first-order channels, the placemarks were placed approximately where the gully domain ended and the (ephemeral) channel domain began (as evidenced by presence of water and/or sand in the channel) (Fig. 1b). Although badlands are formed by numerous gullies, their tremendously high drainage density precludes identification of individual gully systems (Fig. 1c). Denuded hillslopes form when, due to lateral coalescence of gullies, an affected hillslope is stripped of its soil cover. So much so that gully channels or networks disappear and only remnant gully heads can be observed (Fig. 1d). As badlands and denuded hillslopes are large areal entities, the placemarks were dropped approximately at the centre of imaginary polygons, which enclosed the feature in question.

Great care was taken to identify all gully erosion features present within the territory of India, although the presence of clouds in images, unavailability of imagery and poor image quality may have resulted in some features remaining unidentified. However, in all but eight of the cases where the latest image was of suboptimal quality, several images were available dating back to 2020 (starting year of our mapping window) and therefore said issues did not create any unavoidable hindrance. On the contrary, at eight locations (all located close to each other in Eastern India), owing to the lack of multitemporal imagery, we could not bypass the limitations posed by one of the abovementioned image-related constraints, but upon verifying with the corresponding topographic maps, we found no gully erosion features at those areas.

### Recording the management status of gully erosion features

**Fig. 2:**
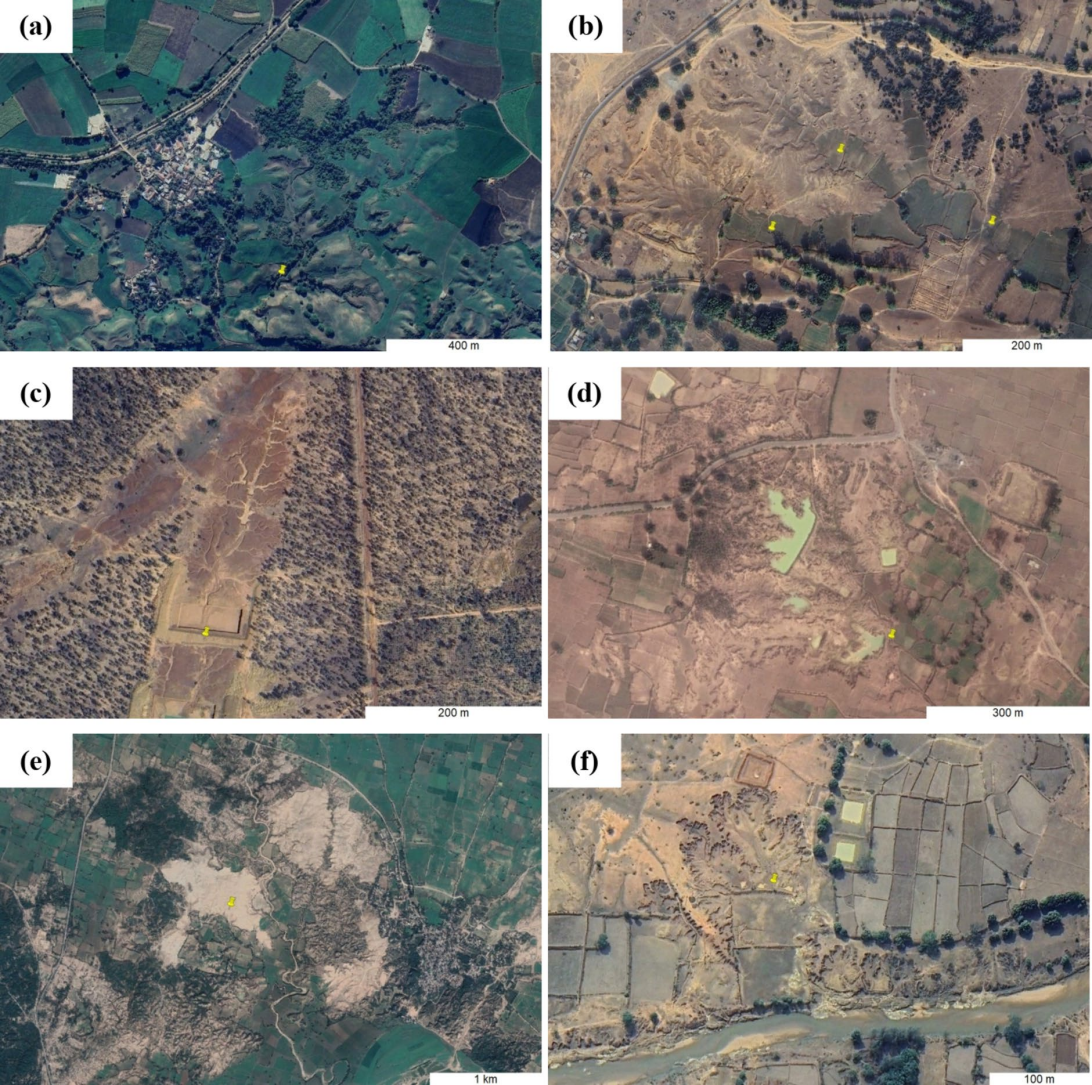
Examples of gully erosion management: (a) gully in-filling for agriculture, (b) gully floor levelling for agriculture, (c) construction of runoff retention pond at the gully catchment outlet, and (d) functioning runoff retention ponds, (e) badlands reclamation and (f) reclamation of a denuded hillslope. *Note: Images exported from Google Earth Pro (2024 Airbus).*

Once all identifiable gully features present within a 15’ cell were mapped, we then re-inspected them to ascertain whether management interventions were present or absent for each. A feature was recorded as ‘managed’ if it was observed to be under reclamation or restoration (partial land levelling in gully systems, badlands and denuded hillslopes to create a surface suitable for agriculture –Fig. 2a, b, e and f) and/or remediation (building check-dams or retention ponds to reduce runoff velocities, trap sediments and ultimately control or stop runoff drainage outside of the gully system – Fig. 2c and d) operations. While reclamation of gullied lands minimises the on-site effect of gully erosion (by reversing the loss of land), building check dams or retention ponds reduces the off-site effects (by preventing the deterioration of water quality and ecology due to sedimentation). Gully erosion features with permanent in-gully vegetation cover were recorded as managed features, but those with only seasonal vegetation coverage (which would not prevent in-situ erosion and/or ex-situ runoff drainage) were identified as unmanaged gully features.

### Estimation of the land area affected by gully erosion

Upon completion of the point-based mapping, all badlands and denuded hillslopes were visually digitised as polygonal features and the area of each polygon was calculated. In contrast, digitising surface areas of the entire population of gully systems in India was not possible because of the sheer number of features identified (*n* = 48,356; see Results). Consequently, to obtain an estimate of the areal coverage of gully systems, we first digitised the individual extents of 500 (~ 1%) randomly sampled gully systems and calculated their average surface area. We subsequently used this information to obtain an estimate of the total gullied area across India as well as in individual states and districts by multiplying the average gully surface area value with the number of gully systems identified in the entire country and its states and districts, respectively.

Confidence intervals indicate measurement precision and the likelihood that a population parameter falls within a limit of values, based on different levels of significance, *viz.*, 90%, 95% or 99%. For a given sample size, the interval will be wider for a higher significance level and vice versa, which helps to understand the uncertainty associated with a sample size. To provide a measure of uncertainty of the obtained gullied area estimates, we calculated the 95% confidence interval of the average gully surface area (sample mean) and used its upper and lower limits (possible limits of the population mean) (Table 1) to determine an area estimation range separately for the country, states and districts. Finally, the total gully erosion affected area (summation of badlands, denuded hillslopes and gully systems’ areas) across India and in separate states and districts was calculated.

**Table 1:** Salient descriptive statistics of surface areas of 500 randomly sampled gully systems.

Min (ha)	0.07
Max (ha)	102.05
Mean (ha)	3.708
Standard deviation (ha)	8.32
Lower 95% confidence interval limit of mean (ha)	2.979
Upper 95% confidence interval limit of mean (ha)	4.437

### Gully management priority assessment

To conduct a quantitative assessment of gully management priority and categorise districts and states into different priority classes, we combined information from the mapped data on current gully affected area and gully management status. For each state and district separately, we calculated two statistics: (i) the relative proportion of the area affected by gully erosion (total gully erosion affected area divided by the total area of a state/district), and (ii) the relative proportion of unmanaged gully erosion features (number of unmanaged gully erosion features divided by the total number of gully erosion features located within a state/district). We then normalised both these statistics by using the respective highest values (yielding relative statistical indices representing gully affected area and gully management status separately for the districts and states with the maximum value being 1), which were subsequently averaged. The averaged normalised statistic provided a quantitative measure representing gully management priority for the districts and states, with lower values indicative of lower gully management priority and vice versa. Finally, five levels of management priority categories (Very high, High, Moderate, Low and None) were obtained through a standard deviation (SD) classification (with one SD intervals around the mean) of the management priority statistic.

## Results

### Gully erosion in India: Spatial distribution and affected land area

**Fig. 3:**
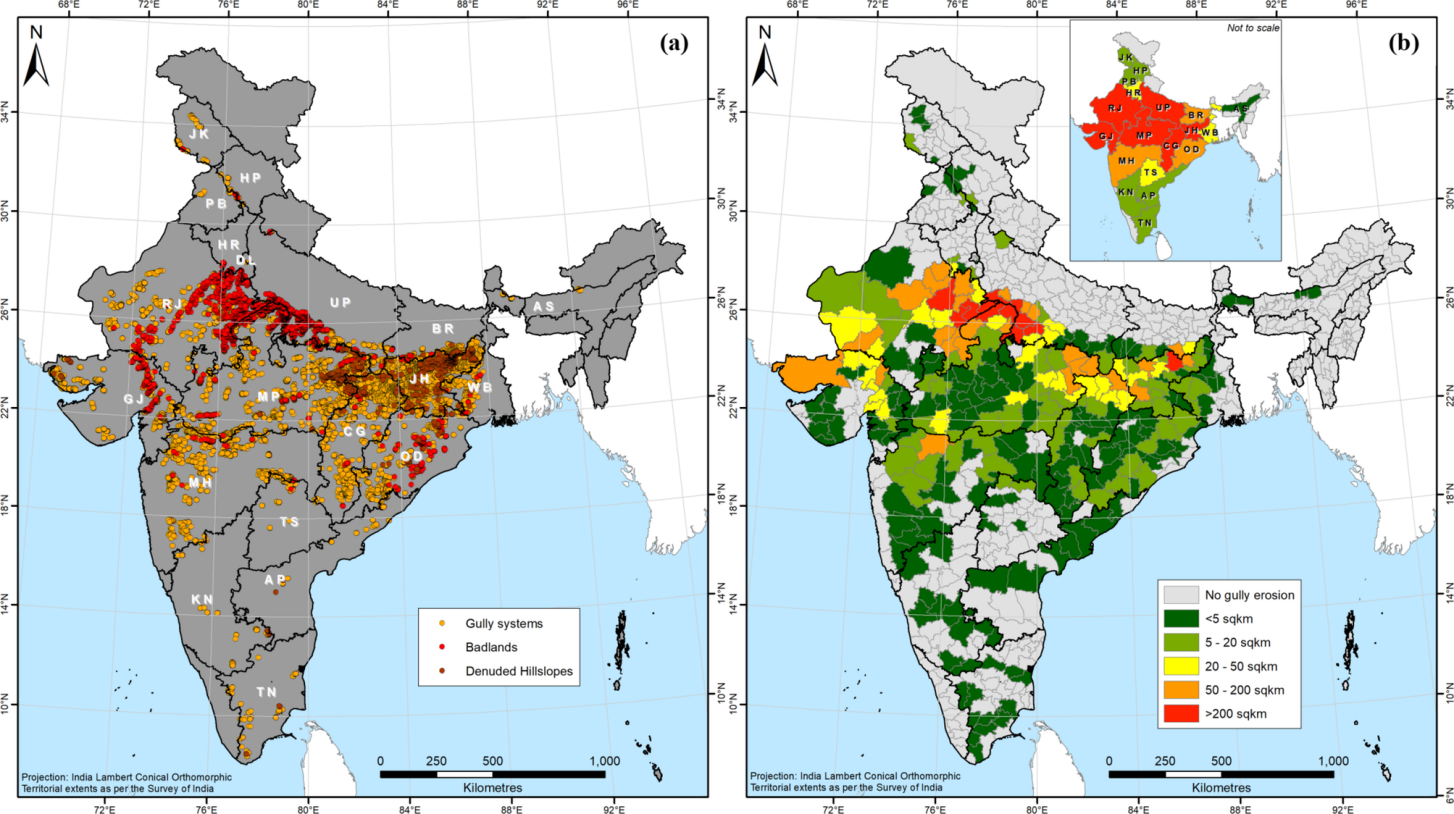
(a) The spatial distribution of gully erosion features in India, (b) Areal extent of gully erosion in India’s districts and states in km^2^ (sqkm). *Note: Maps produced using ArcGIS Pro 3.4. See supplementary information file for details.*

**Fig. 4:**
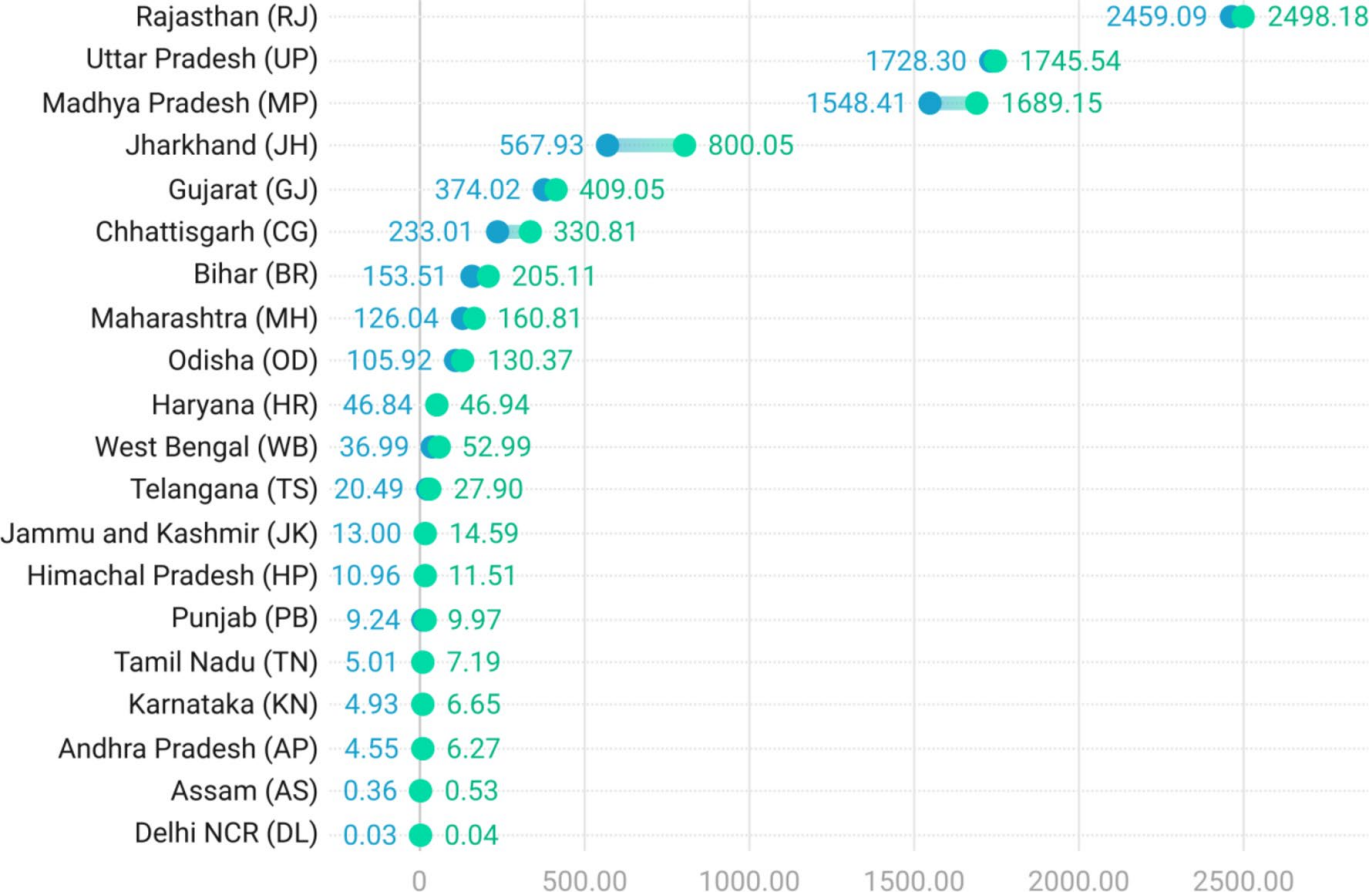
Range diagram showing state-wise estimates of gully erosion affected area (km^2^).

Our mapping has revealed the presence of gully erosion features in 19 of India’s 28 states and the National Capital Region (NCR) of Delhi (Fig. 3). Across 286 districts located within these 20 provinces, a total of 51,755 gully erosion features were identified, of which 48,356 are gully systems, 2,567 are badlands, with the remaining 832 being denuded hillslopes (Fig. 3a). Although the gully systems are the most frequently encountered gully erosion features across India, the areal coverage of the badlands (5,714 km^2^) is the greatest. Using a random sample (*n* = 500) of gully systems (Table 1), it can be estimated with 95% certainty that the total areal coverage of gully systems in India ranges between 1,440 and 2,146 km^2^, while the areal extent of the denuded hillslopes is a considerably lesser 297 km^2^. Therefore, the total gully erosion affected area in India can be estimated between 7,451 and 8,157 km^2^. Although gully erosion only affects 0.24% of the vast land area of India, a marked spatial concentration is observed in the states of Rajasthan, Uttar Pradesh, Madhya Pradesh, Jharkhand, Gujarat and Chhattisgarh (Figs. 3b and 4). While the total area of the mentioned states is *ca.* 38% of Indian territory, they account for a staggering 92% of the total land area affected by gully erosion in the country.

There exists a clear geographical difference between the west and the east in terms of the relative dominance of the mapped gully erosion features (Fig. 3a). The badlands are predominantly located in the states of Rajasthan, Uttar Pradesh, Madhya Pradesh and Gujarat in Western and Central India (Fig. 3a). The total gully erosion affected area in these states is almost four times as much as that of the other states taken together (Fig. 4), even after more than five decades of badlands reclamation^72,73,81^. Rajasthan, Uttar Pradesh and Madhya Pradesh have previously been identified as the states that are worst affected by gully erosion^60,61^, and our area estimates align with this understanding (Fig. 4). In contrast, the gully systems and denuded hillslopes are overwhelmingly concentrated in the eastern states of Jharkhand, Bihar, Odisha and Chhattisgarh (Fig. 3a). In fact, the widespread occurrence of gully erosion in Eastern India was hitherto completely unknown, despite numerous data-driven gully occurrence mapping studies having been undertaken in this part of India within the past five years^60^.

### Gully management in India: Current scenario and future needs

**Fig. 5:**
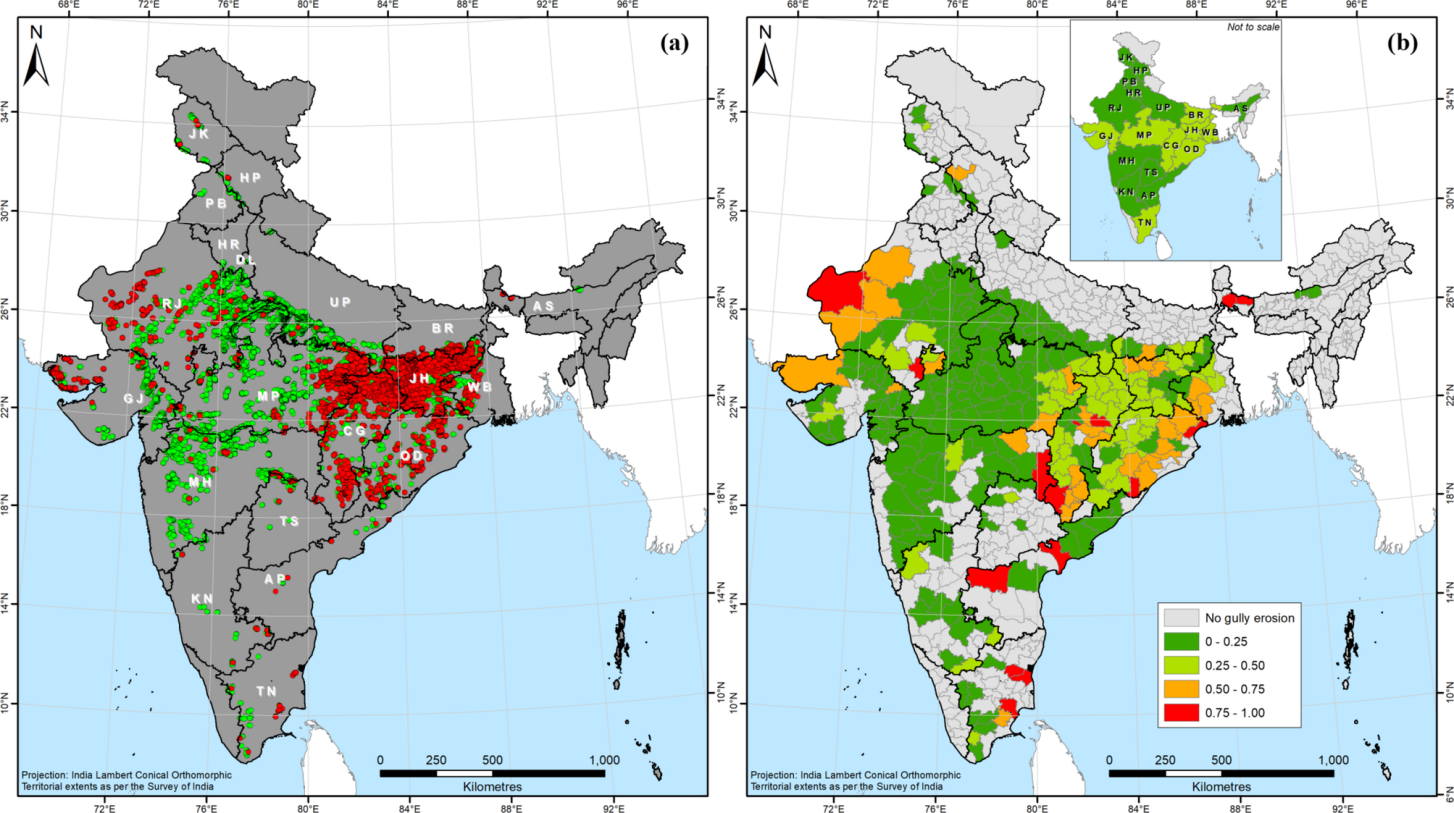
(a) The spatial variation in the management of gully erosion in India (green dot denotes managed and red dot represents unmanaged). Unmanaged (red) points have been placed on top of managed (green) dots for the sake of better visualisation. (b) The relative proportion of unmanaged gully erosion features within each of India’s districts and states. *Note: Maps produced using ArcGIS Pro 3.4. See supplementary information file for details.*

Among the 48,356 gully systems identified through our mapping efforts, 65.4% (31,641) were found to be managed, with a similar proportion (64.5%) of the mapped denuded hillslopes observed to be partially restored. On the other hand, 96.5% of the badlands were found to have been undergoing reclamation operations. Approximately a third of all the mapped erosion features were identified as unmanaged, with the vast majority (98%) of these being gully systems. There is a clear spatial variation in gullied land management in India, with the major proportion of unmanaged gully erosion features observed in Eastern India (Fig. 5). The extent of gullied land is undeniably higher in the badlands regions of Western and Central India (Fig. 3b), but the absence of any land management practices in a significant portion of gully systems in many eastern districts of Madhya Pradesh and across the eastern states of Bihar, Jharkhand, Chhattisgarh, Odisha, and West Bengal is of concern (Fig. 5b). In as many as 80 districts within this eastern region of India, more than a quarter of the identified gully erosion features are not managed, while there is a net excess of unmanaged gully erosion features in 31 of them (Fig. 5b).

**Fig. 6:**
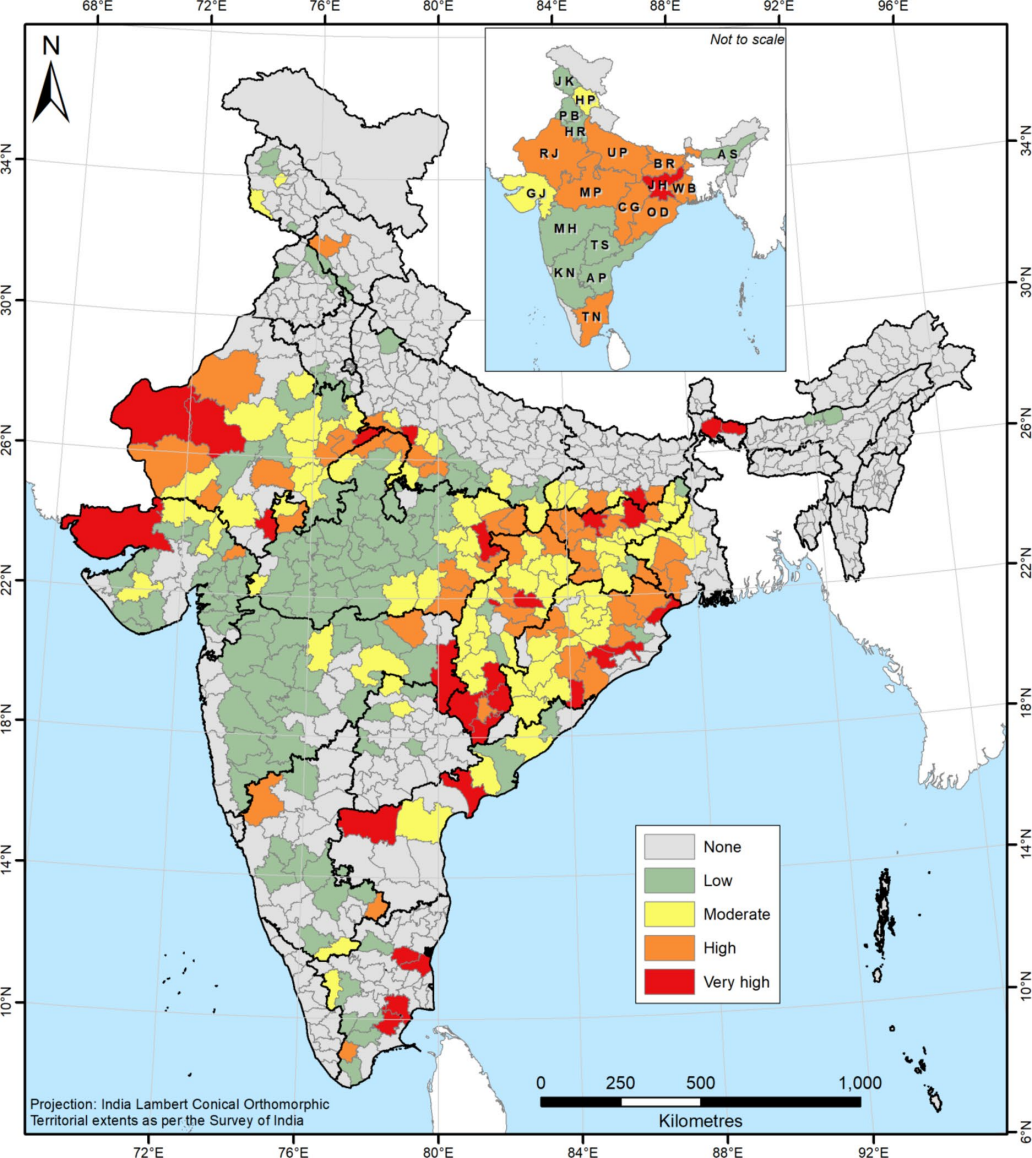
Gully management priority status of India’s districts and states. Priority categories are based on the standard deviation (SD) classification with one SD intervals. *Note: Map produced using ArcGIS Pro 3.4. See supplementary information file for details.*

**Fig. 7:**
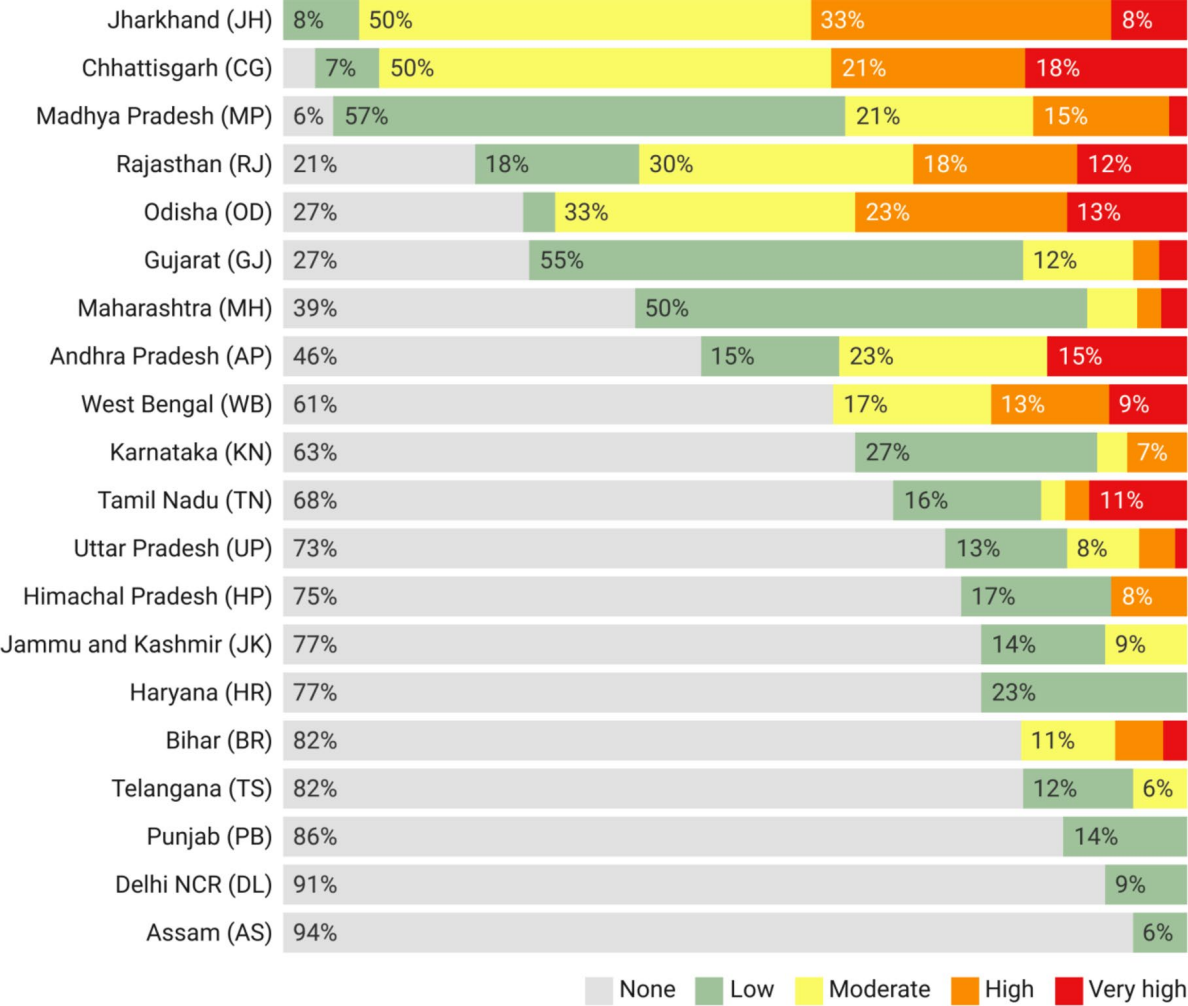
Share of districts with various gully management priority status in the states of India that are affected by gully erosion.

Through our analysis, we have identified a total of 77 districts across India where the management of gully erosion should be prioritised (very high and high priority districts – Fig. 6). Due to the omnipresence of badlands and resultant higher gullied areas, a total of 10 districts across Rajasthan, Uttar Pradesh and Madhya Pradesh have been ascribed very high and high gully management priority status (Fig. 6), despite long-term land reclamation operations having been common in this region^72,73,82,83^. However, more than 60% of the districts classified under the very high and high priority categories are located in Eastern India. The fact that gully erosion in this part of the country is overall more concerning and thus needs most urgent land management attention than that of the badlands is truly an unexpected insight lent by our analysis (Figs. 6 and 7). Beyond the boundaries of the well-known badlands-affected states of Rajasthan, Uttar Pradesh and Madhya Pradesh, the eastern states of Jharkhand, Chhattisgarh, Odisha, Bihar and West Bengal also face a serious gully erosion problem today (Figs. 6 and 7). When the overall district-wise gully management priority of the states is considered (Fig. 7), the eastern states of Jharkhand and Chhattisgarh are the highest ranked, followed by Madhya Pradesh and Rajasthan. The spatial dissonance of the gully erosion hazard in India is best underlined by the fact that Jharkhand in Eastern India is the only state that requires the maximum priority on gully management going forward (Fig. 6).

Few districts lying along the western border of India are also widely affected by unmanaged gully erosion and have consequently been identified as districts where gully erosion management must be prioritised (Figs. 4 and 5b). However, the actual land degradation impact of gully erosion in these districts is likely to be limited as it is a naturally arid and desolate region. Gully erosion once severely affected the Himalayan and Sub-Himalayan states, mainly in response to anthropogenic stressors like deforestation, overgrazing and slash-and-burn agriculture^60^, but it no longer represents a pervasive land degradation threat in these locales (Figs. 5b and 6). Although previous research has almost completely overlooked gully erosion in Southern India^60^, our results suggest that several southern districts, especially in the state of Tamil Nadu, requires urgent land management intervention to tackle the problem of gully erosion therein (Fig. 6).

## Discussion

The uniqueness of India’s gully erosion problem is captured by the fact that while the western parts of the country suffer from the most extreme kind of land degradation perpetrated by gullying, *i.e.*, badlands, despite these having formed due to geological erosion^60^, contemporary gully erosion (since *ca.* 1900) affects Eastern India^84^, with marked differences in spatial extents as well as in land management requirements (Figs. 3, 5 and 6). The extensive impact of gully erosion beyond India’s badlands, especially in the eastern districts and states was previously not known. Our results suggest that gully erosion in Eastern India and to a lesser extent in Southern India represent more serious challenges in the way of India’s LDN-2030 drive than the badlands of Central and Western India (Figs. 6 and 7). While we have found the badlands to account for about 70% of the total gullied area in India, they are now largely stabilised and the gullies therein exhibit limited activity, if at all, which has prompted large-scale land reclamation activities in these regions. However, in the absence of a land management policy^85 ^and resultant lack of institutional oversight, such badlands reclamation operations have often been carried out in injudicious and unsustainable fashion, which have had several unexpected environmental, ecological and socio-economic fallouts^82,83^.

Restoration of gullied lands is known to result in general deterioration of soil (hydrological) properties^86–88^, causing in turn inhibited infiltration, increased hillslope-channel coupling and consequently augmented erosion rates^89–91^. India’s reclaimed badlands suffer widely from high rates of surface soil erosion^92 ^that often exceed local soil loss tolerances^93^, with rills and gullies also forming after intense rainstorms during the monsoons^73,83,94^. If left untreated, these incipient gullies naturally expand and begin to undermine the restoration efforts^92^. Nevertheless, the reclaimed badlands patches are often infertile and result in low crop productivity when farmed^92^. In addition, badlands reclamation has created land ownership disparities between socioeconomic classes and has also had negative ecological impact such as the disappearance of indigenous plant species and loss of habitat for wild animals^82,83^. The need to increase the share of cultivable land in India is understandable, considering it is the most populous country on the planet, but badlands reclamation is overall not a sustainable land management practice.

While we could not explicitly record gully activity as pre-2020 historical imagery of India was not available on Google Earth Pro during our mapping period^95^, it can generally be assumed that a gully system is active if morphological attributes such as abrupt headcuts and sharp sidewalls can be observed from the imagery. Gully erosion in Eastern India is of particular concern, not only because of the marked spatial concentration (Fig. 3a) or that a sizeable proportion of the gullies are unmanaged (Fig. 5), but also because we observed that many gully systems in this part of the country appear to be actively eroding. The fact that Eastern India widely suffers from active gullying possibly explains why most of the gullies are unmanaged, as actively expanding gullies tend to be very unstable and therefore notoriously difficult, if not impossible to control^2,28^. However, in the absence of any mitigative intervention like check dams or runoff storage ponds, the off-site effects of gully erosion in Eastern India are also likely to be severe, particularly sedimentation of rivers and its resultant impact on riparian biota, which have previously been reported from this part of India^60^. Moreover, that gully erosion in this eastern region of India has laid bare the bedrock of entire hillslopes by removing the soil cover is also a finding of great concern and bears further testament to the generally high level of gully activity in Eastern India.

Therefore, India must contend with a double-headed gully erosion problem to successfully achieve LDN by 2030; appropriate rehabilitation of the badlands and effective gully erosion remediation. Fortunately, many pilot studies have been conducted by the badlands research centres of the Indian Institute of Soil and Water Conservation to identify the efficacies of various badlands rehabilitation strategies, including options to maximise ecosystem services, boost carbon sequestration and improve productivity (crop, fuel, fodder) through agroforestry-based practices that ultimately benefits the local communities^60,95,96^. Although a similar body of research on gully remediation in other parts of the country does not exist, the general techniques of gully rehabilitation and prevention are well known^29^.

Gully erosion is usually observed in less than 1% of the land area of regions and countries^32,97 ^and our results confirm that India is no exception in this regard. The numerically minuscule areal coverage of gully erosion owes to a strong spatial variability in gully occurrence patterns, controlled by its well-known threshold-dependent nature^1,5,84^. Despite occupying little areas, gullies cause disproportionately high soil loss in locales where they are spatially concentrated, which is always associated with a host of in-situ and ex-situ effects^3,98^. Gullies are not only extremely difficult to manage effectively when undergoing active expansion^2,28,29^, it is also very challenging to rehabilitate or reverse the land degradation caused by gully erosion primarily because it essentially alters the topography of the affected area, which underscores the scale of the challenge that that lies ahead for the civil administration of the identified 77 high and very high management priority districts and the respective state governments.

## Conclusion

Precise spatial data is critical to the management and rehabilitation of the spatially variable land degradation caused by gully erosion. There exist several comprehensive local and regional inventories of gully occurrence^99–102 ^but country-wide systematic inventorying of gully erosion has only been attempted once before, in a small nation like South Africa^32^, which is indicative of the inherent difficulty associated with such endeavours, especially for large countries. Therefore, our work, which is centred around the first comprehensive gully (management) mapping effort at a sub-continental scale, represents a significant advancement regarding the knowledge of gully erosion and its management in India, as well as presents a mapping methodology that can feasibly be employed elsewhere. Our spatial inventory can further be developed by mapping the various types of gully management measures adopted across India (as illustrated in Fig. 2), and it also paves the way for an unprecedented country-wide gully activity assessment. Although precise delineation of all the identified gully systems in India is difficult, the estimates on their area, which we have expressed using 95% confidence interval of a random sample of 500 digitised gully systems, can further be refined by increasing the sample size. Our database will also serve as high-quality input data (because of its comprehensiveness) in future data-driven predictive gully mapping projects.

The states of Rajasthan, Uttar Pradesh, Madhya Pradesh, Gujarat, Jharkhand and Chhattisgarh (Figs. 3b and 4) account for 38% of Indian territory. However, 92% of the total land area affected by gully erosion in the country lie within these states. Badlands are the dominant gully features in Rajasthan, Uttar Pradesh, Madhya Pradesh and Gujarat, while gully systems predominate in the eastern states of Jharkhand and Chhattisgarh. Contrary to the popular belief that Indian badlands represent the worst-case scenario of gully erosion, we have found that gully erosion in Eastern India poses a more serious impediment to land degradation neutrality in India than the badlands of Central and Western India. However, considering that imprudent badlands reclamation practices have often had several detrimental environmental, ecological and socioeconomic fallouts^82,83^, appropriate badlands rehabilitation is also necessary in regions where they are predominant.

India therefore urgently needs a land management policy that not only shows an appreciation for the overall scale of its gully erosion problem, but also recognises the fundamental differences between badlands and gullies in terms of geomorphology and evolutionary dynamics as well as the range of their social and environmental impact. As agroforestry-based restoration is considered to be widely implementable, an agroforestry policy formulated in 2014 constitutes one of the cornerstones of India’s LDN mission^76,103^. The importance of a complementary land management policy, which outlines a strategy to combat against and remedy various types of land degradation^85^, including but not limited to gully erosion, is obvious. However, considering that gully erosion rates are slated to increase further due to higher rainfall intensities induced by climate change^5^, gully rehabilitation should be accorded maximum importance in India’s LDN plans. In this context, our results can feasibly provide an impetus to pertinent policymaking debates at the provincial and/or national level. As and when India develops a new land management policy or reforms existing land use policies to include land management provisions, our spatial inventory and district-level gully erosion maps will be useful steering instruments in the targeted management of its gullied lands.

## Electronic supplementary material

Below is the link to the electronic supplementary material.

Supplementary Material 1

## Data Availability

The research data will be made available by the first and corresponding author upon reasonable request.
